# Different Strokes for Different Folks? Examining a New Measure for Age-Relative Sexual Attitudes

**DOI:** 10.1093/geroni/igab046.2312

**Published:** 2021-12-17

**Authors:** Allyson Graf, Kinsey Bryant-Lees, Tracy Cohn, Maggie Syme

**Affiliations:** 1 Northern Kentucky University, Highland Heights, Kentucky, United States; 2 Radford University, Radford, Virginia, United States; 3 Hebrew Senior Life, Boston, Massachusetts, United States

## Abstract

Recent research suggests increasingly permissive attitudes towards sexual activity in later life. Harboring more conservative beliefs especially as one reaches older age, however, may translate into how one views and navigates sexual health changes. A sample of participants (N = 706; Mage = 52.72 years, SD = 9.57, range = 36-79; 60.8% male) was recruited through Amazon’s Mechanical Turk to complete a survey on sexual beliefs about age and aging. Participants completed two versions of the Relative Sexual Attitudes Scale (RASA), wherein they were prompted to consider either “someone their own age” or “an older person” in response to items assessing sexual attitudes. Multi-group confirmatory factor analysis was used to confirm the original five-factor structure, reduce the total items from 31 to 25, and establish measurement equivalence for the 36-54 year-old and 55+ year-old samples. Through a series of profile analyses investigating each subscale, scores did not significantly differ between the two prompts, although significant age-group differences were found with the 36-54 year-old age group reporting more open attitudes than the 55+ year-old age group across all subscales, except the traditional mores subscale. Sexual attitude subscale scores also differed by gender, engagement in partnered sexual activity, and whether one had spoken to a health professional about their sexual health in the past year. The findings support use of the RASA for adults 36 and older and highlight applications to understanding differences in sexual health into later life.

